# The association between grade of coronary heart disease and risk of developing keratopathy: a nationwide cohort study

**DOI:** 10.7150/ijms.101733

**Published:** 2025-01-01

**Authors:** Chia-Yi Lee, Shun-Fa Yang, Jing-Yang Huang, Chao-Kai Chang

**Affiliations:** 1 Institute of Medicine, Chung Shan Medical University, Taichung, Taiwan.; 2 Nobel Eye Institute, Taipei, Taiwan.; 3 Department of Ophthalmology, Jen-Ai Hospital Dali Branch, Taichung, Taiwan.; 4 Department of Medical Research, Chung Shan Medical University Hospital, Taichung, Taiwan.; 5 Department of Optometry, Yuanpei University of Medical Technology, Hsinchu, Taiwan

**Keywords:** coronary heart disease, superficial keratopathy, age, infectious keratitis, epidemiology

## Abstract

**Purpose:** To evaluate the association between coronary heart disease (CHD) severity and the risk of developing keratopathy.

**Method:** A retrospective cohort study was conducted with data from the Taiwan National Health Insurance Research Database (NHIRD). A total of 593100, 593100 and 296500 patients were included in the control, mild CHD and severe CHD groups, respectively. The primary outcomes were the development of superficial keratopathy and infectious keratitis with antibiotic usage. Cox proportional hazard regression was used to calculate the adjusted hazard ratios (aHRs) and 95% confidence intervals (CIs) for the primary outcomes among the groups.

**Results:** A total of 30697, 32134, and 15977 superficial keratopathy episodes and 6021, 6010, and 2982 infectious keratitis episodes were recorded in the control, mild CHD, and severe CHD groups, respectively. The incidence of superficial keratopathy was significantly greater in the severe CHD group (P = 0.037), and both groups presented a greater risk of developing superficial keratopathy than did the control group (both P < 0.05). The cumulative incidence of superficial keratopathy was also significantly greater in the severe CHD group than in the mild CHD group (P < 0.001). In the subgroup analyses, the incidence of superficial keratopathy was significantly greater in severe CHD patients than in mild CHD patients older than 70 years, and the correlation between CHD severity and superficial keratopathy incidence was significantly greater in those older than 70 years of age (P = 0.002).

**Conclusions:** Severe CHD is related to a greater risk of developing superficial keratopathy, especially in those older than 70 years of age.

## Introduction

Coronary heart disease (CHD) is a disease characterized by coronary arterial obstruction and cardiac ischemia 1. People with mild CHD experience a relatively chronic course, and medications such as antiplatelet and antilipidemic drugs are often used 2, 3. Nevertheless, severe CHD can result in fatal coronary arterial occlusion; thus, surgical management is usually advocated to prevent death 4. Percutaneous coronary intervention (PCI) can be used to expand the occluded coronary artery in severe CHD patients, and PCI results in improved survival rate 5, 6.

In addition to the heart and coronary vasculatures, CHD may increase the incidence of certain diseases 7-9. According to an earlier study, periodontitis is correlated with the formation and severity of CHD 10. Furthermore, Behcet's disease is a systemic inflammatory defect that increases CHD risk and other cardiovascular impairments 11, 12. On the other hand, CHD is significantly correlated with metabolic syndromes, including hypertension, hyperlipidemia and type 2 diabetes mellitus 13, 14. With respect to the relationship between ophthalmic diseases and CHD, a higher risk of open angle glaucoma was found in CHD patients 15.

Corneal diseases, such as superficial keratopathy, present frequently in people and can result in irritation and ocular pain 16. The severe form of corneal disease, infectious keratitis, can corrupt the corneal structure and contribute to subsequent eyeball rupture and loss of vision 17. Among the causes of keratopathy, dry eye disease is a common etiology for the development of ocular surface damage and superficial keratopathy 18, and viral infection and immunological dysfunction can also lead to the development of superficial keratopathy 19, 20. With respect to the etiology of infectious keratitis, microorganism invasion is the fundamental component of infectious keratitis, which includes bacterial, fungal, protozoan, and viral infections 21. Contact lens wear, ocular trauma and ocular surgery could contribute to corneal insult and the development of infectious keratitis 17. Nevertheless, few studies have evaluated the correlation between CHD status and the risk of developing keratopathy. In addition, the association between CHD severity and subsequent dry eye disease has been illustrated in previous research 22. Thus, the severity of CHD may influence the severity of subsequent keratopathy, which warrants further investigation.

Consequently, the objective of the current study was to survey the potential correlation between CHD severity and the risk of developing keratopathy of differing severities. The general health status of each participant was also included in the analyses. With respect to the terms and criteria for classification in the current study, superficial keratopathy referred to patients who received superficial keratopathy-related ICD-9 and ICD-10 diagnostic codes; infectious keratitis/keratopathy referred to patients who received infectious keratitis-related ICD-9 and ICD-10 diagnostic codes and antibiotic treatment; mild CHD referred to patients who received CHD-related ICD-9 and ICD-10 diagnostic codes and medical treatments; and severe CHD referred to patients who received CHD-related ICD-9 and ICD-10 diagnostic codes and PCI management.

## Materials and Methods

### Ethics declaration

The current study adhered to the Declaration of Helsinki and later amendments. The current study was also approved by both the National Health Insurance Administration of Taiwan and the Institutional Review Board of Chung Shan Medical University (Project code: CS1-20108, date of approval: 06/16/2020). The requirement of obtaining written informed consent was waived by the above two bureaus.

### Data source

The Taiwan NHIRD saves the medical records of 23 million Taiwanese individuals from January 1, 2000, to December 31, 2020. The available records in the NHIRD include the International Classification of Diseases Ninth Revision (ICD-9) diagnostic code, the International Classification of Diseases Tenth Revision (ICD-10) diagnostic code, sex, age, socioeconomic status, place of residence, laboratory codes, image codes, medical department codes, procedure or surgical codes and international ATC codes for all medicines in the Taiwan National Health Insurance System.

### Participant selection

A retrospective cohort study was performed, and patients were defined as having CHD occurrence if these conditions were met: (1) CHD was diagnosed according to ICD-9/ICD-10 codes from 20142019; (2) complete blood cell count, cholesterol, white blood cell differentiation count, high-density lipoprotein, triglyceride, low-density lipoprotein, cardiac angiography and electrocardiogram data were available before CHD diagnosis; (3) age ranged from 20 to 100 years; and (4) participants were regularly followed for more than three months. The index date of the current study was set at 6 months after CHD diagnosis. Additionally, the following exclusion criteria were utilized: (1) demographic data were unavailable, (2) CHD patients died before the index date, and (3) outcomes developed before the index date. To survey the correlation between CHD severity and the risk of developing keratopathy, CHD patients were divided into mild CHD patients who received other medical treatments and severe CHD patients who underwent PCI. One severe CHD patient with PCI was matched to two mild CHD patients with other medical treatments via the propensity score-matching (PSM) method, which was used to consider the distribution of age and sex between the severe CHD patients who underwent PCI and the mild CHD patients who received other medical treatments. Additionally, the mild CHD population mentioned above was matched to the non-CHD population at a 1:1 ratio. After the whole process, 593100, 593100 and 296500 patients were enrolled in the control, mild CHD and severe CHD groups, respectively.

### Main outcomes

The main outcomes in this study were superficial keratopathy and infectious keratitis. Patients with superficial keratopathy met the following criteria: (1) received superficial keratopathy-related ICD-9 and ICD-10 diagnostic codes, (2) underwent slit-lamp biomicroscopy before superficial keratopathy was diagnosed according to the procedure code, and (3) superficial keratopathy was diagnosed by an ophthalmologist. In addition, infectious keratitis was defined as (1) the receipt of infectious keratitis-related ICD-9 and ICD-10 diagnostic codes, (2) the arrangement of slit-lamp biomicroscope tests before the infectious keratitis diagnosis according to the procedure code, (3) the receipt of topical antibiotic eyedrop or ointment prescriptions after the infectious keratitis diagnosis, and (4) the infectious keratitis diagnosis was performed by an ophthalmologist. Only superficial keratopathy and infectious keratitis events that emerged after the index date were considered as the primary outcomes in the current study.

### Confounding factors

Multiple demographic factors, systemic diseases and medications were included in the statistical model of the current study to adjust for the influences of possible confounders of keratopathy development. These covariates include age, sex, hypertension, diabetes mellitus, hyperlipidemia, ischemic stroke, rheumatoid arthritis, systemic lupus erythematosus, Sjogren syndrome, corticosteroids, nonsteroidal anti-inflammatory drugs, and aspirin. The presence of these covariates was determined via demographic codes, ICD-9/ICD-10 diagnostic codes and ATC codes in the NHIRD of Taiwan. To affirm that the existing periods of these covariates in the current study are sufficient to impact the risk of keratopathy, only the covariates that had been established for more than one year before our index date were included in the current study. The people in the current study were followed until keratopathy occurred, they withdrew from the National Health Insurance System, or the end date of the Taiwan NHIRD study (set as December 31, 2020).

### Statistical analysis

SAS version 9.4 (SAS Institute Inc., Cary, NC, USA) was used for the statistical analyses in the current study. Descriptive analyses were performed to display the demography, systemic diseases and medications of the control group, mild CHD group and severe CHD group, and the standardized mean difference (SMD) was used to compare the distributions of initial characteristics. A SMD value greater than 0.1 was defined as a significant difference. Cox proportional hazard regression was then used to calculate adjusted hazard ratios (aHRs) with 95% confidence intervals (CIs) for superficial keratopathy and infectious keratitis among the groups. The potential effects of demography, systemic disorders mentioned in the “confounding factors” section, and medications were adjusted in the Cox proportional hazard regression. A Kaplan‒Meier curve was generated to display the cumulative probability of superficial keratopathy and infectious keratitis between the two CHD groups, and the log-rank test was used to evaluate the cumulative probability between groups. With respect to subgroup analyses, CHD patients were stratified by age and sex, and Cox proportional hazard regression was used to compare the aHR and 95% CI of superficial keratopathy and infectious keratitis between the severe CHD population and the mild CHD population with different ages and sexes. Additionally, an interaction test was performed to explore the effects of CHD severity on keratopathy severity in different age and sex subgroups. Statistical significance was set as P < 0.05, and P values lower than 0.001 are indicate as such (P < 0.001).

## Results

The initial characteristics of the three groups are available in Table 1. The sex ratio was identical among the groups due to the PSM procedure (both SMD = 0.000), and the age distributions of the people in the current study were grossly similar among the groups (both SMD = 0.001). In addition, the ratio of systemic diseases was not significantly different between the two groups (all SMDs < 0.1), and the percentage of patients receiving medication was also not significantly different between the mild CHD group and the severe CHD group (all SMDs < 0.1), except for the ratio of aspirin use (SMD: 0.105) (Table 1). In addition, the rates of hypertension, diabetes mellitus, hyperlipidemia, and aspirin use were significantly lower in the control group than in the mild CHD group (all SMD > 0.1) (Table 1).

**Figure 1:**
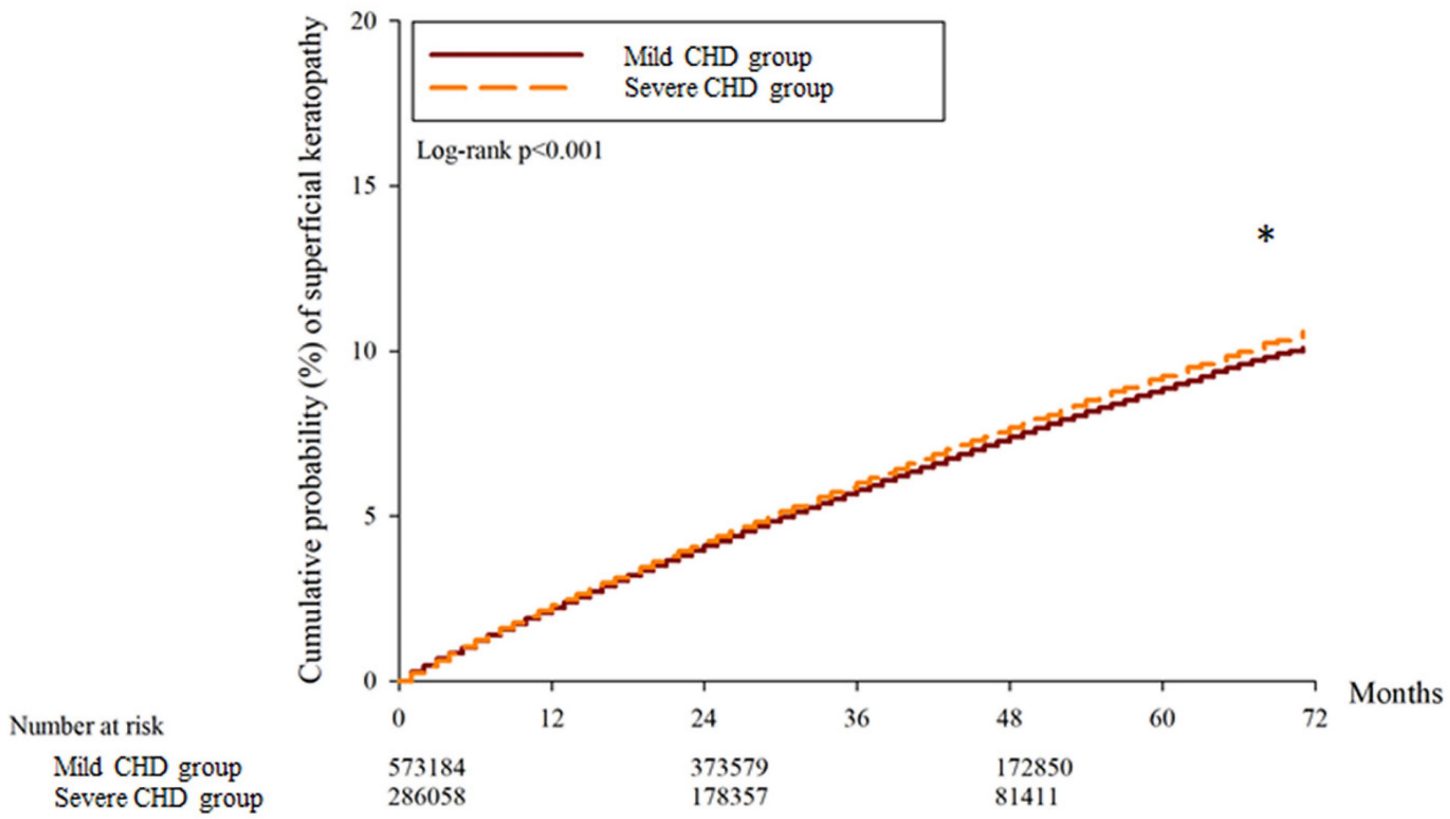
The cumulative incidence of superficial keratopathy between the two groups. CHD: coronary heart disease. * Denotes a significant difference between the two groups.

**Figure 2:**
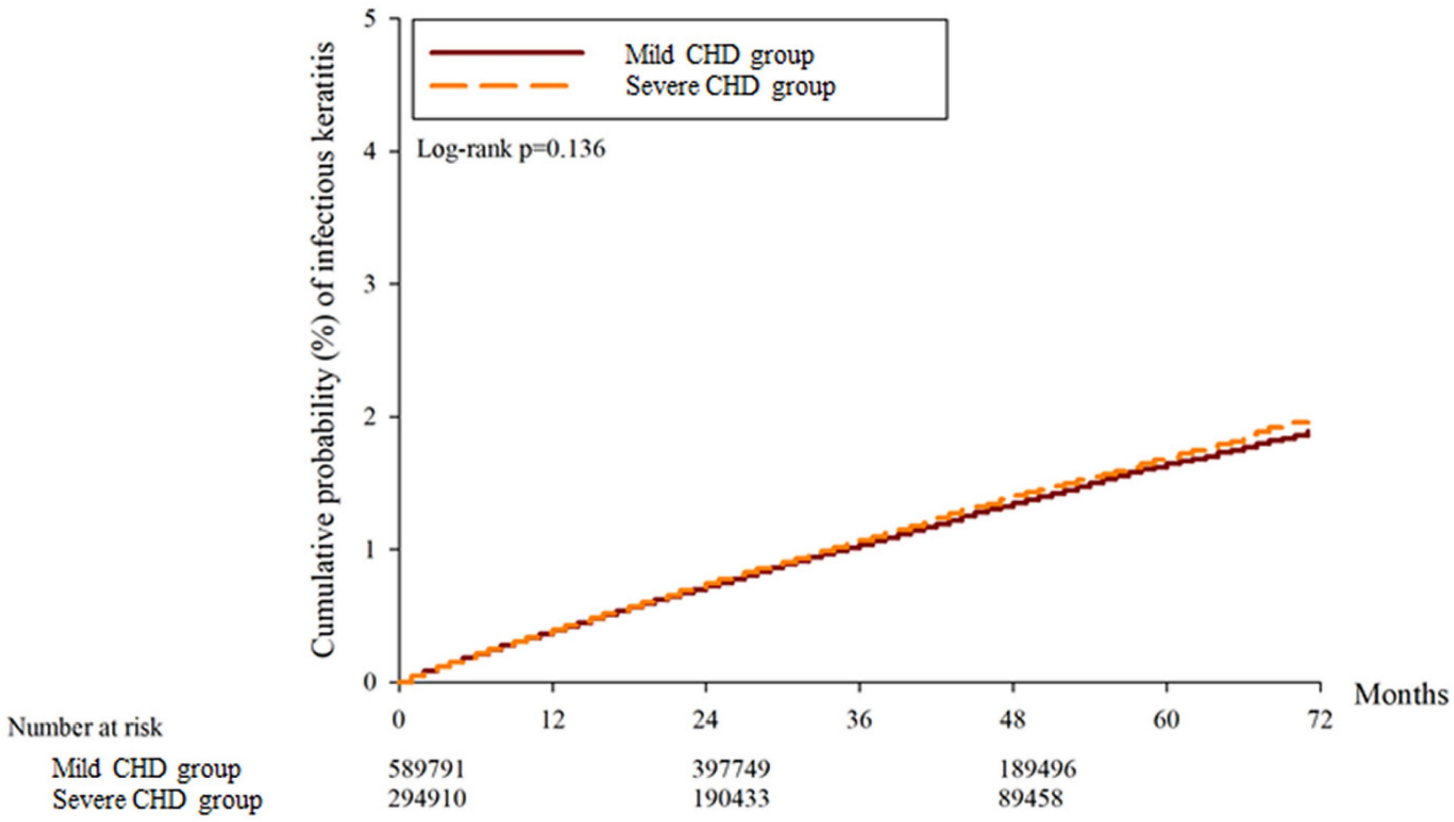
The cumulative incidence of infectious keratitis between the two groups. CHD: coronary heart disease.

A total of 30697, 32134 and 15977 superficial keratopathy episodes were recorded in the control group, mild CHD group, and severe CHD group, respectively. In addition, 6021, 6010 and 2982 infectious keratitis episodes were recorded in the control group, mild CHD group and severe CHD group, respectively. After multiple covariates were adjusted, the incidence of superficial keratopathy was significantly greater in the severe CHD group than in the mild CHD group (aHR: 1.026, 95% CI: 1.005-1.047, P = 0.037), and both groups presented a greater risk of developing superficial keratopathy than the control group did (both P < 0.05). On the other hand, the incidence of infectious keratitis in the severe CHD group was not significantly greater than that in the mild CHD group (aHR: 1.027, 95% CI: 0.979-1.077, P = 0.246), and both groups presented a similar risk of infectious keratitis to the control group (both P > 0.05) (Table 2). The cumulative incidence of superficial keratopathy was significantly greater in the severe CHD group than in the mild CHD group (P < 0.001) (Figure 1), and the cumulative incidence of infectious keratitis was similar between the severe CHD group and the mild CHD group (P = 0.136) (Figure 2).

In the sex subgroup analyses, the incidence of superficial keratopathy was significantly greater in females with severe CHD than in females with mild CHD (aHR: 1.043, 95% CI: 1.011-1.076), whereas the incidence of infectious keratitis did not differ between the sex subgroups (Table 4). The correlations between CHD severity and keratopathy incidence in different severe CHD subgroups were not significant (both P > 0.05) (Table 4). With respect to the age subgroup analysis, the incidence of superficial keratopathy was significantly greater in severe CHD patients than in mild CHD patients older than 70 years (aHR: 1.067, 95% CI: 1.033-1.102) (Table 4), whereas the incidence of infectious keratitis was not significantly different across the severe CHD subgroups. The correlation between CHD severity and superficial keratopathy events was significantly greater in those older than 70 years (P = 0.002), and the correlations between CHD severity and infectious keratitis events were similar across all age subgroups (P = 0.752) (Table 4).

## Discussion

In the present study, severe CHD status was associated with a greater incidence of superficial keratopathy than mild CHD status. Moreover, this correlation was more prominent in patients older than 70 years than in the younger population. On the other hand, the associations between CHD severity and the incidence of infectious keratitis were not significant in the overall population.

CHD can damage coronary arterial vessels to a large extent and can also cause damage to other human tissues 8, 23, 24. CHD is highly related to the development of systemic inflammatory diseases, including ankylosing spondylitis and inflammatory bowel disease 25, 26. Inflammatory status is common in people with CHD, and the neutrophil-to-lymphocyte ratio is a crucial inflammatory marker that can predict the development of subsequent CHD 27. Additionally, the plasma concentrations of the cytokines interleukin and C-reactive protein are significantly greater in people diagnosed with CHD 28. In addition to inflammation, hyperlipidemia is another CHD pathophysiology that is significantly associated with the presence of atherosclerotic plaques and subsequent coronary artery stenosis 29. Atherosclerotic plaque and subsequent CHD could result from high triglyceride expression 30, and higher LDL expression is also a well-established predisposing factor for CHD formation 30. In addition to hyperlipidemia and inflammation, high oxidative stress is another mechanism for CHD that is significantly elevated in those with CHD and acute cardiovascular events 31. With respect to the corneal aspect, oxidative stress contributes to ocular surface damage, and erosion of the corneal surface may occur 32. Moreover, the inflammatory reaction is a critical factor in the development of dry eye disease 33, and severe dry eye disease can cause corneal lesions 34. Infectious keratitis is caused mainly by microorganisms, and a prominent inflammatory response is also found in patients with infectious keratitis 17. Since both CHD and keratopathy have similar mechanisms involving inflammation and oxidative stress 17, 23, 31, 32, we speculate that the severity of CHD may influence the development of subsequent corneal disorders of different forms. The hypothesis was supported by the outcomes of the current study, at least to some degree.

Severe CHD was associated with a greater possibility of subsequent superficial keratopathy in the current study. In previous studies, keratopathy has been reported in patients with systemic inflammatory diseases such as psoriasis 35. Nevertheless, the potential correlation between CHD severity and the risk of developing keratopathy with different severities was not reported in a previous study. To our knowledge, this may be a preliminary experience that reveals a positive correlation between CHD severity and subsequent superficial keratopathy occurrence. Furthermore, only superficial keratopathy episodes that occurred 6 months after CHD diagnosis were counted in the current study; thus, the time sequence between severe CHD and superficial keratopathy could be established. On the other hand, some predisposing factors for corneal disease, including rheumatic arthritis and Sjogren syndrome, were included in the Cox proportional hazard regression to adjust their effect on the development of keratopathy 36. Consequently, severe CHD may be an independent and crucial risk factor for subsequent development of superficial keratopathy. Although the number of keratopathies in the severe CHD group was greater than half of that in the mild CHD group, the mild CHD group had 2-fold more participants and 2.1-fold longer follow-up than did the severe CHD group. Thus, the incidence of keratopathy per person during a fixed interval was significantly greater in the severe CHD group according to the Cox proportional hazard regression models, which were adjusted for the effects of several keratopathy-related risk factors. We believe that this result could present the real risk degree rather than the direct comparison of numerical values. In addition, the cumulative probability of superficial keratopathy was significantly greater in the severe CHD population than in the mild CHD population. This result may further imply the influence of severe CHD on corneal health and that a long-term severe CHD course results in additional damage to the cornea. In contrast, the incidence and cumulative probability of infectious keratitis did not increase in the severe CHD population compared with the mild CHD population. A possible explanation is that microorganism invasion still accounts for the major and prominent pathophysiology of infectious keratitis 17; thus, the effects of inflammation and oxidative stress in infectious keratitis may not be significant.

In the subgroup analyses, females with severe CHD presented with a significantly greater risk of developing superficial keratopathy than women diagnosed with mild CHD did. On the other hand, the male population with severe CHD did not have a significantly greater risk of developing superficial keratopathy than men diagnosed with mild CHD did. A possible explanation for our finding might be that the actual CHD severity in the female population might be greater than that in the male population 37. The criteria for severe CHD are based on the PCI approach, but the patients who underwent PCI did not have the exact same CHD severity. As a consequence, the actual CHD severity in the female population may be greater; thus, elevated inflammatory and oxidative stress leads to a greater incidence of subsequent superficial keratopathy. However, the interaction test revealed that the correlations between superficial keratopathy and severe CHD were similar in different sexes, which further implies that female sex may be a nonprominent risk factor for the development of superficial keratopathy in the severe CHD population. On the other hand, the severe CHD population older than 70 years presented a significantly greater risk of developing subsequent superficial keratopathy development than did mild CHD patients of the same age. Moreover, the correlation between CHD severity and the risk of developing superficial keratopathy in patients older than 70 years was significantly greater than that in their younger counterparts. Few studies have investigated this association. Age is a prominent predisposing factor for the development of corneal disease 38; thus, our findings support the established concept. Severe CHD was not strongly related to keratopathy in any of the subgroup analyses, which is consistent with the results of the whole-population analysis.

With respect to the epidemiology of CHD, CHD is a major vascular disease that is found in approximately 5-6% of the Caucasian population 39, 40, and the prevalence of CHD may reach 25% in specific regions of East Asia 41. Although the prevalence of CHD has gradually decreased in recent decades, severe CHD that presents with coronary arterial occlusion is still diagnosed in more than 30% of all CHD patients 42, and prominent CHD can result in a noticeable mortality rate in the European population according to earlier publications 43. On the other hand, keratopathy is not an uncommon ocular disease, as it is found in more than 1% of people worldwide 19, 44. Although superficial keratopathy does not usually cause visual impairment, such as that observed in patients with infectious keratitis 17, ocular symptoms in those with superficial keratopathy may reduce quality of life. Since CHD and keratopathy affect many people and can lead to substantial burdens in daily life, the associations between these two diseases should be emphasized.

There are several limitations in the current research. First, we consumed the claimed data, not the real medical documents, for all the analyses of the current study. Multiple critical parameters, including the complete blood count results, lipid profile results, echocardiogram results, cardiac angiography results, details of PCI, postoperative status of PCI, recurrence of coronary arterial occlusion, external eye picture of keratopathy, site and pattern of keratopathy, cause of keratopathy, species of microorganism involved in infectious keratitis, and outcome of medical therapies for CHD and keratopathy, were not evaluated. In addition, the retrospective design of the current study could reduce the homogeneity of the study population, although we applied the PSM procedure to increase the degree of homogeneity. Moreover, the criteria for the PCI indication or the diagnosis of corneal diseases may differ across physicians. Thus, the diagnostic standards for severe CHD, superficial keratopathy, and infectious keratitis may be different in this study, thus reducing the reliability of our results. Additionally, the effect of eyelid dysfunction on keratopathy was not analyzed because many of them were only used in self-paid cosmetic surgeries, and lifestyle risk factors (e.g., cigarette smoking, length of cellphone usage) for keratopathy are nearly unavailable in the NHIRD and thus could not be evaluated. Finally, nearly all the people in the current study were Han Taiwanese, and the external validity of the current study may be diminished.

In conclusion, compared with mild CHD, severe CHD is correlated with the risk of developing superficial keratopathy, especially in people older than 70 years. Furthermore, the risk of developing superficial keratopathy attendance is positively related to the disease duration of severe CHD. Consequently, a periodic ophthalmic exam may be recommended for people with severe CHD to manage underlying keratopathy if it is present. Further large-scale prospective studies to evaluate the relationship between CHD extent and the therapeutic outcome of keratopathy are needed.

## Figures and Tables

**Table 1:** Baseline characteristics of the groups

Characteristic	Control group (N: 593100)	Mild CHD group (N: 593100)	Severe CHD group (N: 296550)	SMD1	SMD2
Sex				0.000	0.000
Male	385840(65.05%)	385840(65.05%)	192920(65.05%)		
Female	207260(34.95%)	207260(34.95%)	103630(34.95%)		
Age				0.001	0.001
<40	16844(2.84%)	15836(2.67%)	7799(2.63%)		
40-49	48338(8.15%)	47745(8.05%)	24762(8.35%)		
50-59	115477(19.47%)	115061(19.40%)	57501(19.39%)		
60-69	177515(29.93%)	179176(30.21%)	88431(29.82%)		
>=70	234926(39.61%)	235282(39.67%)	118057(39.81%)		
Comorbidity					
Hypertension	199993(33.72%)	388125(65.44%)	220574(74.38%)	0.156*	0.025
Diabetes mellitus	106461(17.95%)	183624(30.96%)	124581(42.01%)	0.189*	0.049
Hyperlipidemia	99522(16.78%)	252245(42.53%)	170576(57.52%)	0.362*	0.035
Ischemic stroke	36416(6.14%)	47448(8.00%)	30011(10.12%)	0.015	0.019
Rheumatoid arthritis	6821(1.15%)	6702(1.13%)	3796(1.28%)	0.001	0.001
Systemic lupus erythematosus	831(0.14%)	890(0.15%)	860(0.29%)	0.001	0.001
Sjogren syndrome	7473(1.26%)	7354(1.24%)	4181(1.41%)	0.001	0.001
Comedication					
NSAIDs	310429(52.34%)	344710(58.12%)	178790(60.29%)	0.006	0.002
Corticosteroids	114824(19.36%)	104623(17.64%)	64411(21.72%)	0.002	0.004
Aspirin	122001(20.57%)	225200(37.97%)	178849(60.31%)	0.143*	0.105*

CHD: coronary heart disease, SMD: standardized mean difference SMD1: mild CHD group compared with the control group SMD2: mild CHD group compared with the severe CHD group * denotes a significant difference

**Table 2:** The risk of study events among the groups

Event	Control	Mild CHD	Severe CHD
Superficial keratopathy			
Person-months	19802145	19778459	9457210
Event	30697	32134	15977
aHR1 (95% CI)	Reference	1.071(1.010-1.124)*	
aHR2 (95% CI)	Reference		1.145(1.096-1.207)*
aHR3 (95% CI)		Reference	1.026(1.005-1.047)*
Infectious keratitis			
Person-months	21198706	21024190	10077132
Event	6021	6010	2982
aHR1 (95% CI)	Reference	1.002(0.975-1.030)	
aHR2 (95% CI)	Reference		1.035(0.982-1.088)
aHR3 (95% CI)		Reference	1.027(0.979-1.077)

aHR: adjusted hazard ratio, CHD: coronary heart disease, CI: confidence interval aHR1: mild CHD compared to control aHR2: severe CHD patients compared with controls aHR3: severe CHD compared with mild CHD * denotes a significant difference

**Table 3:** Subgroup analysis stratified by sex

Event	aHR (95% CI)	P for interaction
Superficial keratopathy		0.453
Male	1.014(0.987-1.041)	
Female	1.043(1.011-1.076)*	
Infectious keratitis		0.426
Male	1.042(0.983-1.105)	
Female	1.012(0.935-1.095)	

aHR: adjusted hazard ratio, CI: confidence interval * denotes a significant difference

**Table 4:** Subgroup analysis stratified by age

Event	aHR (95% CI)	P for interaction
Superficial keratopathy		0.002*
age<60	0.976(0.939-1.014)	
age 60-69	1.015(0.979-1.052)	
age ≥70	1.067(1.033-1.102)*	
Infectious keratitis		0.752
age<60	1.040(0.960-1.126)	
age 60-69	1.007(0.924-1.099)	
age ≥70	1.019(0.942-1.103)	

aHR: adjusted hazard ratio, CI: confidence interval * denotes a significant difference

## Abbreviations

CHD: coronary heart disease
PCI: percutaneous coronary intervention
NHIRD: National Health Insurance Research Database
ICD-9: International Classification of Diseases Ninth Revision
ICD-10: International Classification of Diseases Tenth Revision
PSM: propensity score matching
SMD: standardized mean difference
aHR: adjusted hazard ratio
CI: confidence interval
N: number
